# Perspectives and Experiences of Nurses Implementing the Safe Steps for De‐Escalation in Acute Mental Health Units

**DOI:** 10.1111/jpm.70094

**Published:** 2026-01-17

**Authors:** Esario IV Daguman, Jacqui Yoxall, Richard Lakeman, Marie Hutchinson

**Affiliations:** ^1^ Faculty of Health Southern Cross University Coffs Harbour New South Wales Australia; ^2^ Faculty of Health Southern Cross University Lismore New South Wales Australia; ^3^ School of Nursing and Midwifery University of Southern Queensland Toowoomba Queensland Australia

## Abstract

**Introduction:**

Efforts to reduce restrictive practices in acute mental health units require more than operational reform; they also need to give voice to clinicians who implement these changes.

**Aim:**

This paper forms part of a broader evaluation of the *Safe Steps for De‐escalation*, which was aimed at investigating the impact of the *Safe Steps* implementation on the perceived professional quality of life of the nurse participants. This paper also presents a qualitative assessment of the process, aimed at identifying the factors that influence the successful implementation of the Safe Steps from the perspective of the nurse participants.

**Methods:**

Safe Steps is a structured approach for de‐escalation, intended to reduce restrictive practices and promote the development and maintenance of therapeutic relationships, as well as individuals' self‐management. It was implemented in three adult inpatient units in New South Wales, Australia, from March 2024 to April 2025. This paper was nested within a mixed concurrent control study and was informed by a pragmatic and complex intervention research framework. Nurse focus group discussions were analysed using reflexive thematic analysis. Paired measures of compassion satisfaction, burnout, and compassion fatigue before and after one‐year implementation were compared.

**Results:**

Scores after implementation indicated a decline in compassion satisfaction and an increase in burnout, compared to the baseline. Two superordinate themes were identified from seven focus groups, with twenty‐six nurse participants: (i) *de‐escalation is a relational, adaptive, and collective nursing practice*, and (ii) *ecological pressures shape the practice of de‐escalation*. These superordinate themes were developed from seven subordinate themes.

**Discussion:**

A cautious interpretation of the quantitative measures is warranted, given the challenges of obtaining follow‐up responses in busy, under‐resourced inpatient units. The thematic findings suggest that successful implementation depends on the organisational and relational contexts in which interventions are deployed.

**Recommendations:**

Future evaluations of the Safe Steps need to consider extending beyond nurses' relational capabilities to encompass the relational responsiveness of multidisciplinary teams.

## Summary

**Originality:**

- This paper introduces the Safe Steps for De‐escalation as a bottom‐up intervention designed to reduce restrictive practices and promote therapeutic relationships within acute mental health units.
- This paper examines the underexplored domains of nurses' professional quality of life in the context of de‐escalation interventions.

**Significance:**

- The findings elevate the importance of relational capabilities and contextual adaptability in embedding and sustaining therapeutic alternatives to coercion.

**Rigour:**

- This paper was guided by a researcher‐made evaluation and implementation framework, which is rarely used in the evaluation of restrictive practice‐reduction interventions.

## 1 Introduction

Moving mental health services explicitly towards changes that help reduce the use of restrictive practices and permeate the entire culture of a system goes beyond training the workforce to act differently (Barbui et al. 2020). Such cultural change entails understanding how service changes are to be implemented for sustainability that overcomes resistance (Whittington et al. 2023). However, this move can be challenging at a time when there are still limited options beyond de‐escalation, ongoing structural barriers in the physical environment, persistent staffing challenges, and insufficient early training for nurses (Snipe and Searby 2023), all of which play a central role in care and restrictive practice events.

Restrictive practices, such as seclusion, physical restraint, and forced medication, are formally regulated measures that limit a person's freedom, usually justified to establish or maintain a sense of safety (Hempeler et al. 2024). These practices sit at the opposite end of the coercion spectrum from informal psychological pressures, such as threats and inducements, which are also considered harmful to therapeutic relationships and are stigmatising (Potthoff et al. 2022). Both informal and formal coercion can be seen as silencing the experiential knowledge of nurses, particularly when framed as part of a unit's routine (Andersson et al. 2020), institutionalised, and directed. Nurses have a limited role in the application of formal coercion; they are merely instrumental (Lakeman 2000).

Giving voice to people enacting service changes widens the understanding of what supports implementation. This bottom‐up approach is central to transforming mental health services (McGinty et al. 2024). It features prominently in efforts to reduce restrictive practices in acute inpatient units. In Safewards studies, staff participants identified choice and co‐creation as instrumental to programme delivery (Mullen et al. 2022). However, these notions may be idealised, given their development within a distinct UK legal context. In trauma‐informed care studies, flexibility in implementation gave staff a sense of satisfaction as practitioners (Saunders et al. 2023). These findings highlight that satisfaction is closely tied to how service change is seen and experienced on the ground. However, the impact of these service changes on nurses' compassion satisfaction, compassion fatigue, and burnout remains underexplored (see Lobo et al. 2024).

### 1.1 Understanding Intervention Complexity: From Evidence to Values

Using frameworks and theories helps identify which elements of an intervention and its context influence outcomes and sustainability (Datta and Petticrew 2013). When implementation frameworks and theories are used, especially those that are complexity‐informed, the intervention's intended outcome is given primacy, rather than how the intervention is delivered in practice (Hawe et al. 2004). This orientation enables those implementing change to adapt interventions to local contexts without narrowly defining and rigidly standardising any service change. Nevertheless, such frameworks and theories are rarely maximised in evaluating and implementing interventions that reduce restrictive practices in mental health settings (Lantta et al. 2023).

The development of meaning behind emerging experiences and perspectives about interventions is central to their competent delivery. Accessing this meaning may be facilitated when complexity‐informed approaches are also employed, as many coercion‐reduction interventions tend to succeed through their complexity (Allen et al. 2018; Finch et al. 2021; Gaynes et al. 2017). Complex interventions involve interdependent components that influence behaviours, perceptions, and values, and are closely embedded in the contexts in which they are dispensed (Hawe 2015). When aimed primarily at preserving autonomy, complex interventions are said to be driven not just by evidence, but by values of dignity, respect, and human rights (Bodryzlova et al. 2024). This approach underlines that the meaning of a mental health intervention for those employing it can be just as vital as its measurable outcomes.

### 1.2 The Safe Steps for De‐Escalation

A bottom‐up intervention was intended to be exemplified in developing the Safe Steps for De‐escalation, or simply the Safe Steps. This multicomponent intervention initially emerged from the values and wisdom of a mental health clinician. The Safe Steps has been designed to empower nurses to use their authentic voice and promote people's capacity for self‐management through a four‐step de‐escalation approach. The approach was based on evidence for the structure and progression of therapeutic responses in a regional acute mental health unit in New South Wales (NSW), Australia (Daguman, Taylor, Flowers, et al. 2025a). Training on the four steps emphasised core intervention values, including nurses' awareness of context and situation (Daguman, Taylor, Flowers, et al. 2025b). Restrictive practice reviews that focused on nurses' relational capabilities were implemented to help embed these values and were linked to reduced seclusion rate in the acute mental health unit (Daguman, Taylor, Flowers, et al. 2025c). The Safe Steps has been further developed and implemented in several acute mental health units across NSW, with the involvement of Aboriginal Elders and cultural health leads, people with lived experience of mental health service use and of coercive engagement with mental health services, and professionals from different disciplines (see Table 1 for an overview of the intervention's content of care).

**TABLE 1:** Key intervention components of the Safe Steps for De‐escalation.

Component	Description
First component: Structured de‐escalation approach	A four‐step framework guiding the recognition, response, and review of escalating situations in acute mental health units. The approach provides a structured process for nurses to facilitate a safe and supportive interaction in which the person in distress can reflect on their circumstances, be validated, collaboratively determine next steps, and identify actions to sustain positive progress beyond the immediate de‐escalation conversation. A mental health clinician developed this component.
Second component: In‐person training and complementary online modules	Training began with site‐wide, in‐person workshops that introduced the four‐step approach, followed by self‐paced online modules. The modules comprised multimedia content (text, diagrams, audio, and video). They were co‐developed with peer workers, Aboriginal Elders, health leads, and interdisciplinary stakeholders. Module topics included (1) introduction to the framework, cultural reflection, trauma, and emotional dysregulation; (2) collaborative de‐escalation and culturally safe care; (3) emotional intelligence skills and trauma‐informed care; and (4) applying the four‐step framework in real‐time responses.
Third component: Restrictive practice review meetings	Deployed in the third month of intervention implementation, monthly reviews were incorporated into existing monthly in‐service sessions in the unit, lasting up to an hour. Nurse educators presented aggregated de‐escalation log data (collected by nurse participants and containing information on the context, functions of behaviours, and strategies used) to facilitate reflection on de‐escalation practice patterns, celebrate nurses' practice of relational capabilities, and identify opportunities for reducing the use of restrictive practices.

The broad aim of this paper was to make sense of the experiences of nurse participants regarding de‐escalation and the implementation of the Safe Steps, and their perspectives on what would support or inform its successful uptake. An additional aim of this paper was to examine the impact of the intervention on the perceived professional quality of life of nurse participants. It was hypothesised that the measures of nurses' professional quality of life would improve after the intervention's implementation, compared to the within‐group baseline.

## 2 Methods

### 2.1 Design

This paper is part of a broader research project employing a mixed concurrent control study design, integrating qualitative and quantitative data on the impact and implementation of the Safe Steps. This paper addresses the project's second and third objectives through surveys and focus group discussions: (i) to examine the impact of the intervention on nurses' professional quality of life, and (ii) to qualitatively assess the process and understand what influences the intervention's successful delivery (from the perspectives and experiences of nurses), respectively. This paper reports findings in accordance with the relevant guidelines from the Journal Article Reporting Standards for Mixed Methods (JARS–Mixed), specifically the Mixed Methods Article Reporting Standards (MMARS), which address the integration of qualitative and quantitative data (Levitt et al. 2018).

At the heart of the pragmatic implementation and evaluation of the Safe Steps is a researcher‐made framework, scaffolded by the properties of complex adaptive systems (Skivington et al. 2021) and principles of pragmatism (Dolan et al. 2022). This framework was based on an evidence synthesis on restrictive practice‐reduction interventions, suggesting that researchers in the field saw such efforts as shaped by their implementation, research, and broader social, psychological, and legal contexts (Daguman et al. 2024). The researcher‐made framework, the intervention's proposed mechanisms, and the protocol for the broader research project have been communicated openly elsewhere in advance (Daguman, Taylor, Flowers, Owen, et al. 2025).

Between March 2024 and April 2025, the Safe Steps was implemented across three adult inpatient mental health units within three public hospitals in two local health districts in NSW, Australia. The surveys were conducted at the baseline and at T4 (months 10 to 12) of the implementation year, while the focus group discussions took place at T4. Together, the implementation sites have 65 beds for low care and observation needs and 10 beds to support people with higher care and observation needs. There is variation in the multidisciplinary team composition, localised models and cultures of care, the extent of peer work integration, and the use and review of restrictive practices across these units. However, each unit is staffed primarily by nursing professionals from different ethnic backgrounds. Furthermore, these units are situated within a legal context characterised by high rates of involuntary care, with admission through processes consistent with the requirements of the NSW Mental Health Act being strongly linked to involuntary treatment (Corderoy et al. 2024).

Ethical approvals for the project were granted by two human research ethics committees within a local health district (2023/PID00297‐2023/ETH00272) and by a university in NSW (Approval Number: 2023/069). Consent for participation in the survey and focus group discussions was obtained from each participant prior to their commencement.

### 2.2 Participants and Recruitment

A purposive sampling approach was employed for the focus group discussions, with no specific inclusion criteria applied, except that participants had been involved in deploying the intervention since the first time point in the evaluation period. Three to five discussions with around six nurse participants were considered an appropriate number of participants to reach data saturation, based on a review of sample sizes in focus group research (Carlsen and Glenton 2011). An a priori power analysis using G*Power 3 (Faul et al. 2007) indicated that a minimum of 60 nurse survey participants currently working in the implementation units (with a 30% margin for potential exclusions and attrition) is necessary to achieve 95% power to detect a medium effect size (*d* = 0.5) using a one‐tailed paired‐samples *t*‐test at an *α* = 0.05. Recruitment for the surveys and focus group discussions was conducted through in‐service sessions, where nurses were provided with the online link to the surveys and the focus group discussion schedules via their work email accounts.

### 2.3 Procedures

The procedures included administering an online nurse survey and conducting in‐person focus group discussions using a prompt.

#### 2.3.1 Nurses' Survey and the Short Professional Quality of Life

Nurses at implementation sites were invited to complete an online survey, which began with an introduction to the study and access to a participant information sheet. After providing consent, participants were asked to complete nine items of the Short Professional Quality of Life Scale (Short ProQOL; Galiana et al. 2020; The Center for Victims of Torture 2019; see www.ProQOL.org; Stamm 2010), along with an additional standardised measure on emotionally intelligent workplace behaviours and a range of demographic questions. The Short ProQOL is a measure of perceived burnout, compassion fatigue, and compassion satisfaction, with higher scores indicating higher levels of the constructs. Norm‐referenced scores are available elsewhere (Galiana et al. 2022), but were not applied here, as the main aim was to compare groups, rather than to promote participants' personal awareness. The norms were also derived from a different geographical context. The demographic data collected in the survey were reported descriptively, but were not included in the within‐group comparisons. For this paper, only data from the Short ProQOL were reported and analysed. Third‐party processes, including the use of a four‐character code for matching data and an independent information technology officer undertaking the data matching and extraction, were in place to ensure that the authors could not link participants' email addresses with participants' data and identities.

In this paper's dataset, preplanned estimation of the scores' internal consistency and factorial validity was abandoned, given the sample size of the gathered matched surveys (see the Results section). This decision aligned with best practice recommendations to avoid unstable or misleading parameter estimates, as small samples substantially reduce the precision and robustness of internal consistency and factor structure analyses (Rouquette and Falissard 2011). As specified a priori, the analysis proceeded using the known three‐factor structure of the Short ProQOL.

#### 2.3.2 Focus Group Discussions and Discussion Prompt

The first author facilitated the in‐person focus group discussions. However, participants' names were not disclosed to him and were intentionally withheld by nurse educators who organised the discussion. Each discussion started with reminders regarding confidentiality, nurse participants' right to pause or withdraw at any point, respecting other participants' experiences and perspectives, and the objectives of the present paper. Participants were also advised to avoid using people's names during the discussions; however, when names were mentioned, they were redacted from the transcripts before analysis.

A researcher‐developed focus group prompt guided the discussion. The prompt had four main sections: (i) nurses' responses to emotional distress, troubling behaviours, and interpersonal conflict, (ii) therapeutic relationships, (iii) de‐escalation competencies needed to respond to emotional distress, troubling behaviours, and interpersonal conflict and (iv) the Safe Steps implementation. The discussion prompt was made to reflect the context in which the Safe Steps was implemented. Follow‐up questions were shaped by participants' initial responses and informed by the first author's evaluation and implementation framework. These included, but were not limited to, questions concerning autonomy (e.g., What did this approach mean to you personally?), emergence (e.g., How did you experience it in the moment?), feedback (e.g., What changes would you suggest moving forward?), and holism (e.g., How does this fit within your broader experience of managing emotional distress, troubling behaviours, and interpersonal conflict?).

### 2.4 Positionality

This paper was guided by a framework developed by the first author to support the evaluation and implementation of the Safe Steps (Daguman, Taylor, Flowers, Owen, et al. 2025). The framework centres on understanding experiences as they were expressed in the stakeholders' own terms, with recognition of meaning that emerges from within the context of their practice of the Safe Steps. Rather than positioning the authors as external experts who interpret other people's experiences, the authors recognised the value of dialogue and co‐construction in shaping understanding. The authors considered the nurse participants' reflections as having been shaped by the culture, relational dynamics, and broader systemic interactions in which the participants are embedded. These influences were not seen as confounds to be controlled (Åsbø et al. 2024), but as instruments for discerning how the Safe Steps was viewed and enacted. The authors did not aim to estimate inter‐rater agreement, aligning with the paper's qualitative analysis approach, which rejects assumptions of objectivity in favour of meaning‐making through researcher engagement with the data (Braun and Clarke 2023).

The analysis was conducted collaboratively by the authors, who came from diverse cultural, educational, and professional backgrounds. Instead of neutralising the authors' positionalities, the authors acknowledged that their backgrounds shape how the data were understood and interpreted. The authors were not concerned with attributing responses to individuals in these settings. Instead, the emphasis was on patterns and meanings across accounts.

### 2.5 Data Analysis

Quantitative and qualitative data treatment approaches were used in this paper. For the quantitative strand, changes in nurses' measures of professional quality of life over time were assessed through paired *t*‐tests by the first author. Non‐parametric alternatives were used where distributional assumptions were violated. Missing data were planned to be imputed for cases with no more than 20% missing responses. Measures with more than 20% of items missing were to be excluded from the analysis.

For the qualitative strand, reflexive thematic analysis, as outlined by Braun and Clarke (2006), was adapted to guide the interpretation of focus group discussion data. The first author undertook the analysis in five iterative stages: (a) re‐engaging with the data, (b) organising, (c) coding, (d) streamlining codes, and (e) developing and refining themes. First, transcripts were re‐read to establish familiarity with the data and identify segments that captured the experiences of nurse participants in implementing the Safe Steps. This initial engagement sought to identify the meanings emphasised within the nurse participants' accounts. Second, the data were brought into order using a spreadsheet. Third, preliminary codes were created, based on the five core areas of the first author's complexity‐informed framework. Fourth, these codes were reviewed, refined, and grouped into broader topic categories that captured recurring and salient aspects of experience, as described by the nurse participants. Finally, these categories were further clustered and condensed into overarching themes. The broader analysis team then revised the refined themes.

## 3 Results

The following section is divided into two parts: the first presents the quantitative survey results, and the second outlines the qualitative themes derived from the focus group discussions.

### 3.1 Nurse Survey Demographics and the Short Professional Quality of Life Outcomes

Fifty‐five participants completed the surveys at baseline. However, only 10 had matched survey data at the final time point and were retained for analysis. The remaining unpaired responses were then excluded. At an individual measure level, no missing responses were noted. Table 2 presents the demographic information of the baseline participants; however, only the most frequently endorsed demographic information is noted for participants with matched survey data to preserve their anonymity.

**TABLE 2:** Key characteristics of nurse survey participants.

Response item	Baseline		With matched survey (*n* = 10)
	*n*	% (out of *N* = 55)	
Sex			
Female	22	40	^a^
Male	10	18	
Prefer not to say	23	42	
Language spoken at home			
English	17	31	
French, Filipino, German, Hindi, Mandarin, Nepali, Vietnamese	14	25	
Prefer not to say	24	44	^a^
Identifies as Aboriginal and/or Torres Strait Islander			
No	53	96	^a^
Prefer not to say	2	4	
Country of birth			
Australia	33	60	^a^
China, India, Italy, Nepal, the Netherlands, the Philippines	20	36	
Prefer not to say	2	4	
Ethnicity			
Oceanian	25	45%	^a^
Southern and Eastern European, Western European, North African and Middle Eastern, Southeast Asian, Northeast Asian, Southern and Central Asian	27	49	
Prefer not to say	3	5	
Highest educational attainment			
Hospital‐trained, diploma	5	9	
Bachelor's degree	39	71	^a^
Graduate certificate/Diploma, coursework master's, master's research	10	18	
Prefer not to say	1	2	
Has a specialist mental health qualification			
Yes	11	20	
No	35	64	^a^
Prefer not to say	9	16	
Has permanent employment			
Yes	50	91	^a^
No	3	5	
Prefer not to say	2	4	
Year(s) in the mental health nursing practice space			
Less than a year	12	22	
1–3 years	18	33	
3–5 years	5	9	
5–10 years	10	18	^a^
More than 10 years	8	15	
Prefer not to say	2	4	
Year(s) working in the current unit			
Less than a year	18	33	
1–3 years	16	29	
3–5 years	4	7	
5–10 years	9	16	
More than 10 years	7	13	
Prefer not to say	1	2	

^a^ Given the very small sample for the matched surveys, only the most frequently endorsed response for a specific demographic variable is shown to preserve participant anonymity. ‘Prefer not to say’ was a nonresponse option in nurse surveys that allowed participants to opt out of answering a survey item without providing a reason.

**FIGURE 1:**
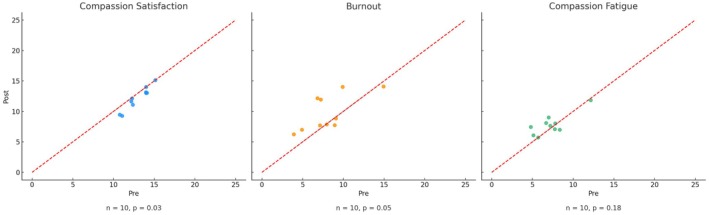
Brinley plots illustrating individual paired scores on the three subscales of the Short Professional Quality of Life: Compassion satisfaction (left), burnout (centre) and compassion fatigue (right). The Brinley plots depict baseline scores on the *x*‐axis and post‐implementation scores on the *y*‐axis. Points on the diagonal line indicate no change. Points above the diagonal line indicate the increase in scores post‐implementation, while those below it show the decrease in scores post‐implementation.

The Short ProQOL scores and comparison outcomes were tabulated in Table 3. Based on normality assumption checks, the burnout and compassion fatigue scores were normally distributed, supporting the use of paired *t*‐tests. In contrast, compassion satisfaction scores violated normality and were analysed using the Wilcoxon signed‐rank test. However, the exact *p*‐value for the Wilcoxon signed‐rank test could not be computed due to tied and zero difference scores. Instead, an asymptotic *p*‐value with continuity correction was reported, indicating that some participants showed no change in their scores or had identical pre‐ and post‐scores (see Figure 1). Ultimately, a significant decline was observed in the compassion satisfaction scores, accompanied by a significant increase in the burnout scores. Since the scores from the three measures following the intervention did not significantly improve compared with those at baseline, this paper's hypothesis is not supported.

**TABLE 3:** Comparison of the nurse participants' Short Professional Quality of Life scores.

Measure	Time point	*M*	SD	Mdn	IQR	Test statistic	*p*	*M*_diff_	95% CI	*d*
Compassion Satisfaction	Pre	12.90	1.45	13.00	2.00	*V* = 0	0.03	−0.80	—	−1.01
	Post	12.10	1.97	12.50	1.75					
Burnout	Pre	8.10	3.03	7.50	2.00	*t*(9) = 2.33	0.045	1.70	[0.05, 3.35]	0.74
	Post	9.80	2.94	8.50	4.00					
Compassion Fatigue	Pre	7.30	2.00	7.00	1.75	*t*(9) = 1.46	0.18	0.50	[−0.27, 1.27]	0.46
	Post	7.80	1.75	7.50	1.00					

*Note:* There were only ten participants with matched scores at the final evaluation time point.

### 3.2 Focus Group Discussion Demographics and Thematics

The total number of completed focus group discussions was seven, with 3–5 participants in each session. Each site that implemented the Safe Steps was represented, with one site hosting four discussions and 3–5 participants each, another hosting two discussions and 4 participants each, and the last hosting one discussion with 4 participants. Table 4 presents the age, work roles, highest nursing educational background, time since becoming a nurse, and time of employment in the implementation unit of the focus group participants. Participant demographic data were not reported by site to preserve anonymity. The focus group discussions lasted between 34 and 49 min, with an average duration of 43 min.

**TABLE 4:** Key characteristics of nurse focus group discussion participants.

Response item	*n*	%
Age		
20–39	13	50
40 and above	12	46
Prefer not to say	1	4
Ethnicity		
Oceanian	11	42
Southeast Asian, Southern and Central Asian, African	15	58
Work role		
Enrolled nurse	5	19
Registered nurse	14	54
Clinical nurse specialist, clinical nurse educator, nurse unit manager	6	23
Prefer not to say	1	4
Highest nursing education		
Diploma	5	19
Bachelor's degree	13	50
Graduate certificate/Diploma, coursework masters, master's research	7	27
Prefer not to say	1	4
Year(s) since becoming a nurse		
Less than a year to 9 years	13	50
10 years and beyond	9	35
Prefer not to say	4	15
Year(s) in current unit		
Less than a year to 9 years	14	54
10 years and beyond	7	27
Prefer not to say	5	19

**FIGURE 2:**
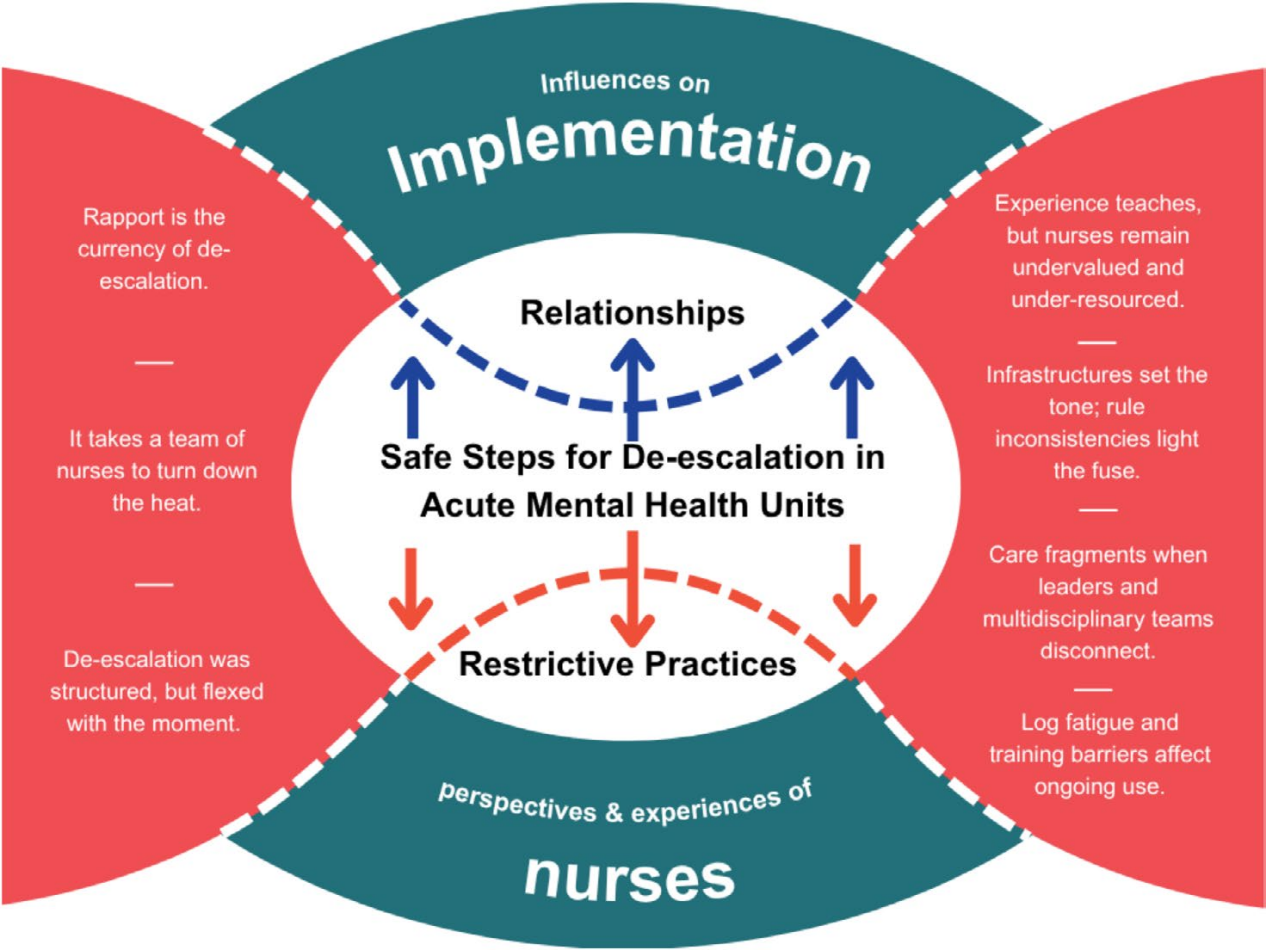
A diagram of the subordinate themes from the reflexive thematic analysis of nurses' focus group discussions on the Safe Steps implementation. Many visual elements in this figure, such as colours, shapes, lines, text content, and text placement, were adapted from an image published in an open‐access research article (Daguman, Taylor, Flowers, Owen, et al. 2025).

The reflexive thematic analysis of the focus group discussions indicated seven themes (see Figure 2), which were further clustered into two superordinate themes. The first, *De‐escalation is a relational, adaptive, and collective nursing practice*, echoes how nurses experienced and deployed de‐escalation in their everyday practice. The second, *Ecological pressures shape the practice of de‐escalation*, represents how wider organisational and environmental backdrops support or constrain de‐escalation and intervention uptake.

#### 3.2.1 De‐Escalation Is a Relational, Adaptive, and Collective Nursing Practice

This theme encapsulates how nurse participants saw, experienced, and enacted de‐escalation, including the human connection, mutual support, and responsive adaptation in the unfolding situation that form the bedrock of therapeutic responding. Three subordinate themes follow and illustrate this foundation.

##### 3.2.1.1 Rapport Is the Currency of De‐Escalation

Participants consistently described rapport as the most effective and humane instrument in de‐escalation. It was viewed as a multifaceted concept, strategy, and philosophy of care, where there is respect, therapeutic connection, and a shared understanding of what makes people safe. Rapport was said to be an essential relational competency built when nurses ‘sit with [the person] … listen to them, and … validate them’ (F7S3). Knowing a person's history, preferences, and early warning signals was also considered a manifestation of rapport‐building, which enables a nurse to intervene without escalating conflict. As one participant noted, ‘When you have a good rapport with a person … [they are] more likely to listen to you than someone who has just walked into the ward [; they do] not know them’ (F1S1).

Rapport was further described as a form of resistance to coercion, which empowers a nurse to challenge pressures to use restrictive practices. One nurse reflected, ‘Even if it means other staff saying, “You should just put them in seclusion” … having the conviction to say no, no, let us try this first’ (F2S1). In the same breath, a nurse is ‘able to maintain a really good rapport with [the] patient, if [they] do not have to hold them down and restrain them’ (F6S1). Small gestures of flexibility, such as meeting simple, reasonable requests, were likewise framed as sustaining rapport. Nevertheless, rapport was also viewed as fragile, subject to emotional distress, trauma, or sudden shifts in a person's state. As one participant remarked, ‘Sometimes, even if you have a good rapport with that patient … they do not even acknowledge you … things can change’ (F3S3).

##### 3.2.1.2 It Takes a Team of Nurses to Turn Down the Heat

Participants noted that responding to events of emotional distress, troubling behaviours, and interpersonal conflict in the unit is a collective undertaking. It is a team's work, where each one ‘treat[s] each other with respect’ (F7S4), or where colleagues ‘encourage … and validate what [they] feel, because everyone has been in the same situation’ (F4S6). Conversely, team division was seen as a barrier to de‐escalation efforts, as people ‘will use that staff splitting skill’ (F1S5). Surveillance‐driven practices, such as reporting colleagues through incident management systems for perceived non‐compliance with dominant unit culture, were also seen as a hindrance to collaboration by one participant.

Some participants specified that de‐escalation often requires role negotiation and the ability to draw on the strengths of ‘who[ever] has got a better relationship with that patient, or even someone from a similar background, similar race’ (F3S1). Strategic team decisions were said to include considerations about experienced ‘sexual assault … trauma‐informed care … [what the person] prefer … [and their] involve[ment] in the treatment plan.’ (F4S4). Gender dynamics and perceived authority also influenced team decisions. One participant narrated, ‘Someone like myself [a male nurse] is not going to be as effective because there is another potential alpha[male] in the room … more effective is sending our smallest girl, a female, to do the de‐escalation’ (F5S2). The ability to step back or step in without ego was also appreciated as an indication of professional maturity. ‘It is not necessarily reflected as a failure on you … You got to say, “okay, I am not being effective” … step back’ (F5S2), a participant remarked.

##### 3.2.1.3 De‐Escalation Was Structured, but Flexed With the Moment

A participant shared that they have engaged with the Safe Steps as ‘a good guideline, but not as a rigid rule.’ (F1S5) However, many noted that existing practices, personal experience, and clinical judgement shaped their framework uptake. Some ‘might not do it one, two, three, four’ (F6S1), similar to a turn‐by‐turn process. Others emphasised that the Safe Steps reflected what nurses in the units were already doing ‘for such a long time’ (F5S2), even ‘prior to being trained’ (F6S3) or ‘before [implementation] started’ (F1S1). A participant felt their agency was taken away by the imposition of yet another perceived policy. Many participants furthered that it did ‘not necessarily change’ (F4S3, F2S1) their practices. On the other hand, some shared that the Safe Steps helped strengthen their ability ‘to identify early signs of aggression’ (F4S3), to be more relational (rather than pharmacological) in their responses, and to ask ‘permission [on whether] you do and you do not’ (F5S2) do certain things. There was also an expression suggesting that the framework served as a form of reassurance, making them ‘feel like … [they are] doing the right thing’ (F2S3).

#### 3.2.2 Ecological Pressures Shape the Practice of De‐Escalation

This theme explores the broader environment in which de‐escalation occurs, including organisational structures, resource limitations, and unit‐level conditions that shape the possibilities for safe and relational care. The four subordinate themes that follow convey these influences through recognition of experiential learning, the role of physical and policy contexts, and the gaps across teams, leadership, and training.

##### 3.2.2.1 Experience Teaches, but Nurses Remain Undervalued and Under‐Resourced

Participants articulated how experiences in high‐pressure environments scaffold the development of mental health nursing competencies. One participant commented, ‘You have to get exposed to the different situations, that is how you get experience’ (F1S2). Unfortunately, this on‐the‐job learning is infrequently recognised within formal education, with ‘new grad[uates having] no experience’ (F6S4). In response, a nurse participant attempted to learn vicariously through ‘great mentors’ (F2S3) at work.

A corollary to issues around experiential learning is the concern that mental health nursing is misunderstood and undervalued. ‘It is not highlighted at uni[versity]’ (F6S4), one participant said while sharing that there is only one subject on mental health nursing in a three‐year bachelor's degree. At work, a nurse participant noted that nurses from outside of mental health services ‘think that [nurses in acute mental health units] sit down on [their] bums and do nothing … [and that others] do not see what [nurses in acute mental health units] actually do.’ (F6S3). This undervaluation of the profession was considered a contributing factor to staffing shortages in inpatient units. One participant relayed the potential connection between these two issues by saying that ‘Out of the graduating class … two of us came in. There [were] about 150. Half of them went to Queensland for better pay, half of them spread out, but two of us stayed in’ (F6S4). In turn, less therapeutic one‐on‐one staff time, weakened nurses' capacity ‘to be proactive … sitting and talking [with the person]’ (F7S3), and burnout were said to have ‘probably increase[ed] … because … staffing has not been great’ (F4S2).

##### 3.2.2.2 Infrastructures Set the Tone; Rule Inconsistencies Light the Fuse

Participants pointed out that the state of physical environments can exert an influence on the escalation or containment of emotional distress, troubling behaviours, and interpersonal conflict. Facilities with ‘wear and tear [that] is not anymore “wear and tear”’ (F4S3), cold temperatures in high observation areas, and the mislabelling of ‘a sensory room … [that] is more like an entertainment room’ (F4S3) were viewed as negatively affecting opportunities for recovery‐focused support. On the other hand, a participant noted that structural disparities influence the quality of care that nurses provide, with one participant saying:

Different ward[s] … is chalk and cheese … [Units with lower total capacity] for patients, [but] have a higher obs[ervation capacity] … discharge probably one person a month … whereas we [with fewer high observation beds per total beds] are forever attempting discharge, discharge to make beds (F6S1)

Participants also pointed out structural disparities in the form of dated, shared‐room layouts, with one advocate for ‘single rooms … because there are a lot of arguments over rooms … and then eventually the whole ward erupts’ (F3S5).

In relation to potential interpersonal conflict, several participants also described daily tensions arising from the inconsistent enforcement of unit policies, particularly regarding smoking and food delivery, due to differing interpretations of rules or misleading upstream messaging. One participant disclosed that ‘in the paramedics, like in the ambulance in e[mergency] d[epartment, people] are told that once they come to our unit, we allow them to smoke, and then we say you cannot smoke’ (F4S6). According to the nurse participants, inpatients perceived these inconsistencies as arbitrary or unfair, which contributed to escalations on the ward. One nurse participant recounted that a person receiving care had said, ‘Oh, how come, like yesterday I was allowed [to smoke], and now I am not’ (F4S6). Another participant shared that ‘someone [got] aggressive [at] 12 o'clock for [food delivery] … [however,] junior staff … [would not see] it as … a big deal, and … so it creates an inconsistency between nurses’ (F1S1).

##### 3.2.2.3 Care Fragments When Multidisciplinary Teams and Leaders Disconnect

In this theme, participants highlighted how inconsistent medical staffing and poor integration of wider team roles undermined effective care and escalation responses. Inconsistent medical staffing was considered a challenge posed by short‐term psychiatrists, who had ‘all been fly in, fly out’ (F6S1; the level of medical seniority or training was unclear). These medical officers were said to have had ‘no investment in the unit’ (F6S1), had made people receiving care ‘feel like they are not being heard’ (F5S2) during medical reviews, and had been seen to ‘run … into the back passage [of a unit during an escalation], and the nurses are left to clean up the mess’ (F6S1). In contrast, participants noted that health security assistants (HSAs or security personnel) are ‘out on the unit a lot and see a lot more of the escalating behaviours’ (F2S1) and ‘actively spend time … as a presence, playing cards, talking [with inpatients]’ (F7S3). However, they were also seen as under‐utilised and need to ‘be just as much involved with [de‐escalation] as [how] nurses are’ (F2S1). Given such dilemmas, some participants recommend that ‘the multidisciplinary teams, [including] the social worker, the doctors, [be] on the same page’ (F3S5) and ‘would benefit [from] tak[ing] a course’ (F6S3) on the Safe Steps.

Participants also expressed frustration with senior management, who were seen as disengaged from the realities of frontline mental health support. Leaders who are at a higher rank than nurse unit managers were perceived as ‘not understand[ing] the complexities … [and] coming down [to the unit] only when something negative has happened.’ (F7S2). In contrast, ‘the status of doing well is actually going to go to management … who really do not give a [expletive] what happens on the ward’ (F6S1), a participant exclaimed. Another participant also pointed out that the culture in their unit, during the implementation of the Safe Steps, had undergone substantial change in line with the recent leadership transitions, which they thought had interrupted the momentum of introducing and sustaining positive change. The participant further noted a lack of genuine buy‐in from some leaders who had only recently transitioned into their roles.

##### 3.2.2.4 Log Fatigue and Training Barriers Affect Ongoing Use

The initial engagement of nurse participants with the Safe Steps was considered strong. However, participants also described how the demands of everyday acute care practice made it difficult to maintain long‐term use. For some, the de‐escalation logs were reflective and helpful in tracking and prompting the demonstration of relational capabilities, with one nurse sharing, ‘Doing the logs … showed me, like, wow, I actually did it a lot of times.’ (F2S3). However, many point out that completing the logs brings them no personal benefits, to the point that it has been deprioritised amid ‘doing all the other paperwork’ (F2S1) and ‘hit[ting] one crisis after another’ (F6S1).

On the other hand, participants also expressed worries about the delivery and limited accessibility of training facilitated by nurse educators. Conflicting schedules, different learning preferences (i.e., shorter, more frequent training sessions spaced across a timeline versus concentrated into a single, all‐day event), and staff turnover were seen as challenges in embedding the framework into routine practice.

## 4 Discussion

This paper reported on the professional quality of life measures of nurse participants from the Safe Steps evaluation. These measures are among the under‐explored nurse‐sensitive outcomes related to de‐escalation training interventions (Price et al. 2024). Findings from the nurse focus group discussions were also reported, which supported the emergence of influences on successful intervention uptake. The Safe Steps is a structured de‐escalation approach, deployed in three adult mental health inpatient units in NSW, Australia, to reduce restrictive practices and promote therapeutic relationships and self‐management.

After the Safe Steps implementation, burnout and compassion satisfaction scores were found to be unfavourable, contradicting this paper's hypothesis. However, with a very small sample size, caution is needed in interpretation. Meaningful inferences from the standardised measures cannot also be drawn, given the inability to estimate the measure's psychometric validity. Using measures with uncertain validity may mean that the impact of a mental health intervention, as evaluated through such measures, could be spurious (Daguman and Taylor 2024). In terms of effect direction, the noted increase in burnout contradicts the reduction seen in other inpatient de‐escalation training interventions (Goodykoontz and Herrick 1990; Paterson et al. 1992; Taylor and Sambrook 2012), including the null finding seen in the City Nurses (Bowers et al. 2006).

These quantitative findings do not necessarily indicate that the Safe Steps was ineffective. However, they portray the professional quality of life measures as distal outcomes, meaning they are influenced by innumerable external factors (Brenner et al. 1995). As indicated in one of the focus group discussion themes, the possible explanation underlying these findings (and perhaps the very small sample size at T4) could be the staffing shortages in the implementation units. This explanation aligns with the findings from a US multicentre survey, which showed that hospitals with insufficient nurse staffing and high workloads had significantly more nurses reporting burnout and job dissatisfaction (Aiken et al. 2023). An additional explanation for these quantitative findings can be seen in the qualitative findings in this paper on unsupportive unit culture and service leadership. These findings are similar to descriptions of organisational cultures, where leaders respond to concerns with avoidance, whether by acknowledging them but taking no action, providing unclear responses, or reacting defensively (Jackson et al. 2013). This avoidant leadership has been considered to erode trust, amplify emotional strain, and increase feelings of isolation, making it challenging for nurses to sustain their resilience over time.

In this light, there is scope to amplify the Safe Steps' capacity to build nurses' resilience, given that emotional intelligence (EI) is already an intervention value and training content. EI can be mechanised to support the development of professionals' resilience and effective emotional self‐management in challenging clinical contexts (Hurley et al. 2020). In the context of the Safe Steps implementation, applying brief, structured coaching sessions about EI and other relational capabilities to existing restrictive practice review meetings could strengthen nurses' emotional awareness and resilience in practice. More importantly, increasing the frequency of restrictive practice review meetings from monthly to bimonthly iterations could potentially improve nurses' emotional responses to the de‐escalation work. However, systemic problems need systemic solutions. When solutions are framed as issues of employees' vulnerability, there appears to be an endorsement of systemic problems as work hazards that employees need to endure, rather than as problems that management needs to address (Virkki 2008). This point is tantamount to saying that the EI skills of female nurses, as seen in a theme in this paper, should not be painted as admirable merely because women are culturally expected to demonstrate them, particularly in containing violence (Baines 2006).

There were two superordinate and seven subordinate themes. A broader understanding of the themes suggests a circular and mutually reinforcing connection between them. For example, (i) the problem of under‐resourcing may reflect enduring systemic issues, such as (ii) the low professional regard for mental health nursing as a specialist nursing subdiscipline. This low professional regard may contribute to a self‐perpetuating cycle of (iii) burnout, (iv) diminished compassion satisfaction, and (v) inadequate systemic investment in staff development and support. There is support for the link between the aforementioned five issues in a systematic review on the perceived impacts of associative stigma against mental health nurses (Njaka et al. 2023). Such stigma needs to be countered, as it may create them‐and‐us beliefs that nurses could internalise and unknowingly translate into othering behaviours (see Daguman and Taylor 2025 for the statistical link between these stigma mechanisms), including restrictive practices. As Lakeman (2023) also opined, the institutional invalidation of mental health nursing, through exclusion from workforce counts, reduced professional recognition, and inequitable access to reimbursement, training, and research funding, could erode the specialty's standing and undermine its workforce sustainability.

The Safe Steps was introduced as a structured approach; however, many nurse participants emphasised the value of adapting it to their own workflows and nursing team dynamics. It appears that the participants exercised some degree of ‘intuition’, which can be understood as the flexible use of EI capabilities to integrate emotional and technical reasoning in contextually relevant ways (Hutchinson et al. 2018). This intuition is not to be considered a deviation from the intended intervention. Instead, it reflects findings from qualitative assessment of process in other mental health and nurse‐targeted interventions (Forsberg et al. 2025; Hunter et al. 2020), implementation science principles of adjusting interventions with real‐world circumstances (Brewer et al. 2024), and the brief psychotherapy ethos of becoming responsive to the needs of people receiving care (Lakeman 2024). This qualitative finding on intuition validates the quantitative cluster analysis findings from the broader research project in which this paper is nested. In 163 days of the intervention implementation, nurse participants demonstrated variable uptake of the four steps in the structured de‐escalation approach, as well as the complementary use of other de‐escalation techniques (Daguman, Yoxall, et al. 2025). However, a note of caution is due. According to Benner's (1982) framework, genuine intuition emerges only when at least 5 years of clinical experience are integrated with specialist education. Can then the intuition suggested by the focus group participants, many of whom are without specialist preparation, truly be considered ‘expert’ judgement, or merely routine competence reframed as expertise?

The Safe Steps intervention reflects a bottom‐up approach. It offers alternatives to restrictive practices within existing resources, without requiring system‐wide transformation through cultural and workforce redesign, as seen in top‐down approaches. This differentiation between different understandings of change has been described in various ways, including first‐ versus second‐order change (Watzlawick 1974), single‐ versus double‐loop learning (Senge and Westendorp‐Kauffmann 1992), and ‘evolutionary’ versus ‘revolutionary’ change (Tushman and Romanelli 1985). However, scaling up the Safe Steps will need participating mental health services to be ‘ambidextrous’ (Tushman and O'Reilly 1996, 8), that is, capable of both sustaining incremental, bottom‐up improvements and obtaining radical, top‐down changes to tackle systemic constraints and embed the intervention across the services. Despite nurses’ valuing of the practice‐validating framework for de‐escalation, many participants faced persistent barriers, as seen in several other themes, that senior service or hospital executives may arguably best influence through permeating organisational changes.

Many themes were centred on the agents, rather than the objects, of change. The themes explored the actions and interactions of senior mental health service management, psychiatrists, health security assistants, fellow nurses, social workers, paramedics, nurse educators, and the individuals receiving care. These themes suggest that sustainable change is not merely decreed through operational redesigns, but often surfaces from ‘relational response‐ability’ (Moriggi et al. 2020, 281): a proactive and situated capacity to attend, respond, and relate with care. From this perspective, service transformation unfolds through emotionally attuned relationships that continually evolve in context, characterised by mutual responsiveness that supports lasting and meaningful change. A more informative and fairer evaluation of the Safe Steps implementation may then require broadening the intervention's target beyond nurses' relational competencies to include the capabilities of multidisciplinary teams for relational responsiveness, especially those with low turnover, cohesive dynamics, leadership that frames involvement as opportunity, and mid‐career professionals (as also suggested by the demographic composition of those who engaged meaningfully in both the matched surveys and focus group discussions).

There appears to be a layered cascade of institutional power. Of the relationships given primacy in the focus group discussions, those between nurses and psychiatrists, as well as nurses and health security assistants, stood out. These kinds of relationships are not new, albeit a saddening displacement of nurses' therapeutic purpose and engagement. The nurse–psychiatrist relationship, as suggested in this paper and elsewhere (Löfström et al. 2025; Sarı et al. 2025), has been characterised by a hierarchy and distance, where nurses are considered tools of medical authority (Lakeman 2000), yet sometimes feel unsupported in moments of imminent risk. In contrast, the nurse participants' perspectives suggest that their sense of safety hinges on the presence of health security assistants, who are considered indispensable in maintaining order during escalations (Ayhan et al. 2025). A quantitative finding from the broader research project in which this paper is nested validates this qualitative finding. The Safe Steps implementation did not alter the rate of Code Black events within or between the compared groups during the one‐year intervention period (Daguman, Yoxall, et al. 2025), suggesting that nurse participants relied on health security assistants for de‐escalating some escalations. Code Black is an emergency code that calls for the presence and support of security guards in response to personal safety threats (Muir‐Cochrane et al. 2020). This qualitative finding is further consistent with what has been said in forensic psychiatry; guards are the ‘executants in order to reach disciplinary or therapeutic goals’ (Holmes 2005, 5). Taken together, psychiatric authority appears to flow through the nurses and then to the security guards. Nurses could be considered the tools of psychiatric power, and security guards can be seen as an extension of the power that nurses themselves possess.

This paper has several limitations. First, a purposive sampling approach was employed to recruit nurse participants from the implementation of the intervention. This approach ensured that those involved were positioned to share relevant perspectives and experiences on implementation. However, this approach may have introduced self‐selection bias, as participants who were more engaged with the intervention or held stronger views were more likely to participate in the focus groups. Second, the focus groups and survey participants were below the planned sample size requirements, and the research was confined to three implementation sites, each with unique contexts nested in a distinct Australian mental health service backdrop. In Australia, there is no specialist mental health nursing preparation pathway (Lakeman et al. 2024), the involuntary hospitalisation rate of public acute inpatient units is the second highest of 22 countries (Sheridan Rains et al. 2019), and the rate of as‐needed psychotropic administration in acute inpatient units is higher than comparable units in the United States, Canada, and Britain (Baker et al. 2008). These limitations restrict the data representativeness and generalisability of findings to other settings. In this light, services with different workforce educational preparation, legal frameworks, and care models may need to make necessary adaptations to their intervention, implementation, and evaluation. Third, using a third‐party four‐digit random code system in the survey may have deterred participation from nurses who had misplaced their unique codes, and consequently, contributed to the poor survey response rate. Future evaluation of the Safe Steps may consider routine measures that could capture specific dimensions of nurses' professional quality of life. Finally, while the larger study intentionally centred on the perspectives and experiences of nurses, the primary target audience of the Safe Steps intervention, giving voices to other key stakeholders, such as lived experience practitioners, health security assistants, and psychiatrists, was not included and may have provided complementary insights into the broader implementation context.

## 5 Conclusion

This paper presented an evaluation of the Safe Steps for De‐escalation in relation to nurses' professional quality of life measures, as well as a qualitative assessment of the process through focus group discussions. The matched measures of nurses' professional quality of life were not favourable to the Safe Steps implementation. However, a potential bias arose from the very small sample size at the final evaluation time point and the consequent inability to estimate the psychometric quality of these measures. The thematic findings shine light on the influence of relationships between agents of change and the broader context in which change was deployed. These thematic findings further suggest that a shared responsibility for positive change across multidisciplinary teams, as well as support from service management and leaders, are among the factors that could sustain the Safe Steps. Success in further implementations of the Safe Steps is more likely when contextual adaptations are given primacy.

## 6 Relevance to Clinical Practice

Minimising restrictive practices requires more than sharpening nurses' technical competencies; it depends on giving nurses a voice in embedding service changes into the everyday practice of acute care provision. The Safe Steps can be one way among many to support nurses in implementing less coercive responses to emotional distress, troubling behaviours, and interpersonal conflict. However, such an approach needs to be accompanied by good support from mental health service leaders and the different professionals within a unit. For nurses, a less coercive nursing practice also appears to be moving beyond being tools of medical authority to becoming active partners in cultivating humane mental health support.

## Author Contributions

**Esario IV Daguman:** conceptualisation, methodology, software, formal analysis, investigation, resources, validation, data curation, writing – original draft, and visualisation. **Jacqui Yoxall:** resources and writing – review and editing. **Richard Lakeman:** resources and writing – review and editing. **Marie Hutchinson:** conceptualisation, methodology, resources, writing – review and editing, funding acquisition, and supervision.

## Funding

This paper is part of a larger study funded by Southern Cross University and the Translational Research Grant Scheme of the NSW Office for Health and Medical Research.

## Conflicts of Interest

Esario IV Daguman is supported by a PhD scholarship jointly funded by Southern Cross University and the Translational Research Grant Scheme of the NSW Office for Health and Medical Research. One funding body provided feedback during the initial protocol development to enhance the study's scientific merit. However, neither organisation was involved in the original conceptualisation, actual conduct, analysis, reporting, interpretation, or writing of this paper. Adjunct Professor Richard Lakeman, who is a co‐author of this manuscript and a member of the *Journal of Psychiatric and Mental Health Nursing* editorial board, had no editorial involvement in this submission and did not participate in its peer review. The perspectives expressed in the Introduction and Discusssion of this paper are those of the authors and do not necessarily reflect those of the funding bodies, the authors' institutional affiliations, or the research and implementation partners associated with the larger project in which this study is embedded.

## Data Availability

The datasets collected, analysed, reported, and interpreted for this study are not publicly accessible, as restricted by the conditions of the ethical approvals obtained.
